# Purinergic signaling modulates endothelial-to-mesenchymal transition in human aortic valve endothelial cells

**DOI:** 10.1016/j.bbadva.2026.100190

**Published:** 2026-04-18

**Authors:** Vera Schmidt, Andreas Weber, Artur Lichtenberg, Jürgen Schrader, Pascal Martsch, Mareike Barth, Payam Akhyari

**Affiliations:** a Department of Cardiac Surgery, Medical Faculty and University Hospital Düsseldorf, Heinrich-Heine-University Düsseldorf, Moorenstraße 5, 40225 Düsseldorf, Germany; b Department of Cardiology and Vascular Medicine, West German Heart and Vascular Center, University Hospital Essen, Hufelandstraße 55, 45147 Essen, Germany; c Department of Thoracic and Cardiovascular Surgery, Medical Faculty, West German Heart and Vascular Center, University Hospital Essen, Hufelandstraße 55, 45147 Essen, Germany

**Keywords:** Endothelial-mesenchymal transition, Purinergic signaling, Human valvular endothelial cells, Ectonucleotidases, Calcific aortic valve disease, Adenosine

## Abstract

Endothelial-to-mesenchymal transition (EndMT) is a phenotypic switch in which endothelial cells acquire mesenchymal characteristics, involving both functional and morphological changes. While EndMT is essential for cardiac development, its aberrant activation contributes to adult cardiovascular pathologies, including calcific aortic valve disease (CAVD). Dysregulation of ectonucleotidases—membrane-bound enzymes that regulate extracellular ATP and adenosine metabolism—has been implicated in such diseases. Altered extracellular nucleotide signaling influences valvular interstitial cell (VIC) degeneration and may interact with valvular endothelial cells (VECs) undergoing EndMT. The objective of this study was to investigate the role of the purinergic signaling system in regulating EndMT in human aortic VECs.

Primary human VECs were cultured in vitro and treated with inhibitors of ectonucleoside triphosphate diphosphohydrolase 1 (CD39), ectonucleotide pyrophosphatase/phosphodiesterase 1 (ENPP1), and 5′-nucleotidase (CD73), alongside adenosine and P2 purinergic receptor agonists. EndMT markers and signaling pathways were assessed via phosphorylation assays and mRNA expression analysis of key transcription factors, including SLUG, SNAIL, ZEB1, and ZEB2. Inhibition of ATP- and AMP-hydrolyzing enzymes (CD39, ENPP1, CD73) enhanced p38 phosphorylation and modulated SLUG expression. Activation of P2 and adenosine A2B receptors altered SNAIL levels, while A2A receptor signaling influenced ZEB1 and ZEB2 expression. These perturbations resulted in pronounced morphological changes consistent with EndMT.

In conclusion, dysregulation of the purinergic signaling system induces EndMT in human aortic VECs, highlighting a potential mechanistic link between extracellular nucleotide metabolism and valvular pathology. Targeting purinergic pathways may represent a therapeutic avenue for CAVD and related vascular disorders.

## Highlights

• Dysregulation of the purinergic signaling system leads to EndMT in VECs.
• First study linking purinergic signaling to EndMT in human VECs.
• Enhanced ATP impact promotes mesenchymal phenotype in VECs.
• Modulation of ectonucleotidase function alters EndMT-associated transcription factor expression in VECs.

## Graphical abstract

**Figure:**
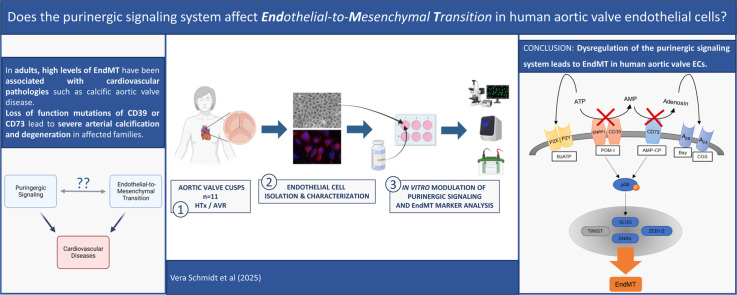

## 1 Introduction

Endothelial-to-mesenchymal transition (EndMT) is a process by which endothelial cells acquire a mesenchymal phenotype and respective functional changes take place. The expression of endothelial markers such as CD31 and VE-cadherin is lost, whereas expression of mesenchymal markers such as α-smooth muscle actin (αSMA) and Vimentin is upregulated [[1], [2], [3]]. Initially, EndMT was identified in embryogenesis where it is necessary for endocardial cushion formation and heart valve development [4] and was considered a special form of epithelial-mesenchymal transition (EMT), a well-studied, similar process in epithelial cells [5,6]. Though crucial during development, in the adult organism high levels of EndMT have been associated with cardiovascular pathologies such as atherosclerosis, cardiac fibrosis or vein graft remodeling [[7], [8], [9]]. Interestingly, lower levels of EndMT seem to persist in the adult valve [10] and it has been hypothesized that this allows a subset of valvular endothelium to replenish the turnover of valvular interstitial cells (VIC) [11]. In valve calcification, activated, mesenchymal-like VIC are present and contribute to matrix remodeling, biomineralization and inflammation [[12], [13], [14]]. In this context, a recent study by Gee et al. demonstrated that valvular endothelial cells (VEC) actively induce pathological remodeling and calcification of VIC via EndMT [15]. Furthermore, Hjortnaes et al. showed that EndMT may precede VEC osteogenesis, whereas VIC inhibit EndMT and osteogenesis in VEC [16]. Collectively, these studies highlight the relevance of EndMT and VEC-VIC interactions in valve pathophysiology, but the regulating factors are yet barely understood.

The purinergic signaling system utilizes purine nucleotides and nucleosides as extracellular messengers and mediates short-term (acute) signaling functions in mechanosensory transduction, secretion and vasodilatation. On the other hand, long-term (chronic) functions of purinergic signaling relate to cell proliferation, differentiation, and death involved in development and regeneration [17]. Released nucleotides are hydrolyzed extracellularly by a variety of cell surface-located enzymes referred to as ectonucleotidases. Ectonucleoside triphosphate diphosphohydrolase 1 (CD39) catalyzes the hydrolysis of extracellular ATP via ADP to AMP, and then ecto-5′-nucleotidase (CD73) dephosphorylates AMP into adenosine [18]. Activity and expression of the key enzymes CD39 and CD73 were shown to be associated with various diseases such as cancer, autoimmune conditions and arteriosclerosis [19,20]. Previous studies from our group reported the critical role of these key enzymes in the degeneration process of VIC and demonstrated enhanced biomineralization by inhibition of CD39 and ENPP1, whereas inhibition of CD73 resulted in protective effects [21].

Following the approach established in the study, the same pharmacological agents were used to investigate extracellular purinergic signaling: To modulate extracellular purinergic signaling, sodium polyoxotungstate (POM-1) was used as an established inhibitor of NTPDase1, NTPDase2, and NTPDase3[22], which has been applied in a variety of experimental settings, including human cell culture models [23]. Inhibition of CD73-mediated conversion of AMP to adenosine was achieved using the competitive ecto-5′-nucleotidase inhibitor α,β-methylene-ADP (AMP-CP), a compound that has been shown to effectively block CD73 activity in human cell culture systems [24,25]. To selectively activate adenosine receptor signaling, we employed CGS-21,680, a well-characterized and widely used selective agonist of the adenosine A₂A receptor, which has been utilized to study A₂A-mediated signaling pathways in vitro, including in human endothelial cell models [26,27]. In addition, BAY 60–6583 was used as a potent and selective agonist of the adenosine A₂B receptor [28], a compound that has already been applied in human endothelial studies to investigate A₂B-dependent signaling [29].

The existing evidence highlights purinergic signaling as well as EndMT as important factors in the physiology and pathophysiology of the cardiac valves, but to date, there are no studies on the relationship between these mechanisms and their interrelation in VEC.

Therefore, we aimed to investigate the potential role of the purinergic signaling system with respect to EndMT induction in VEC.

## 2 Materials and methods

### 2.1 Human VEC isolation

Aortic valve cusps were obtained from 11 patients who underwent either orthotopic heart transplantation or valve replacement (age 60 ± 13 years, range 33–78) at the University Hospital Düsseldorf, Department of Cardiac Surgery, Düsseldorf, Germany. Patients gave informed consent and the research protocol was approved and performed according to the institutional ethics committee (study ID: 2018–298-bio) and the Declaration of Helsinki [30]. VEC were isolated from the obtained tissue samples. Specifically, aortic valve cusps were digested for 4 min in 0.25 % Trypsin-EDTA (Thermo Fisher Scientific) at 37 °C under sustained agitation. Digestion was stopped using equal parts of medium [Growth Medium MV, PromoCell supplemented with 1 % Penicillin-Streptomycin and Amphotericin B (Thermo Fisher Scientific)] and tissue surface was scraped before removal. The remaining suspension was filtered using a 100 µm sterile strainer and centrifuged at 300 x g for 5 min to obtain a cell pellet. Cells were resuspended in fresh medium, seeded onto a 1 % gelatin coated 25 cm^2^ culture flask and kept at 37 °C and 5 % CO_2_ until confluence and further passaging.

### 2.2 Patient data

Patients undergoing aortic valve replacement or heart transplantation (HTx) at a single center between October 2018 and May 2021 were considered for inclusion in this study. During this period, a standardized collection of aortic valve tissue was performed intraoperatively following a predefined protocol. Tissue harvesting was carried out by a trained research team in close collaboration with the cardiac surgery department. Clinical data, including demographic characteristics, comorbidities, previous surgical interventions, medication, and laboratory values, were collected for all donors to allow reliable interpretation of the experimental results. Only samples with sufficient tissue quality for endothelial cell isolation and downstream analyses were included in the final study cohort.

### 2.3 Magnetic cell sorting

Isolated cells at passage 1 were positively selected for CD31 by magnetic cell separation using LS columns (Miltenyi Biotech). In brief, isolated cells were detached from the culture flask using 0.25 % Trypsin and incubated with CD31 magnetic microbeads according to manufacturer’s instructions (Miltenyi Biotech). The obtained cell suspension was passed over a separating column and CD31-positive cells were eluted in the final step. Purified cells were further cultivated on a 1 % gelatin coated 75 cm^2^ culture flask and kept at 37 °C and 5 % CO_2_ until confluence and further passaging. Endothelial cells at passage 3 were cryopreserved and stored in liquid nitrogen until use in experiments.

### 2.4 Immunocytochemistry

Purified VEC at passage 3 were cultured on 1 % gelatin coated glass cover slips (G. Menzel) until confluency and subsequently fixed and stained. In brief, cover slips were washed twice with PBS and fixed in 4 % paraformaldehyde (Roth) for 10 min at room temperature. The fixed cells were incubated with 0.25 % Triton X-100 (NeoLab) in PBS, followed by 1 hour incubation with 5 % serum albumin (Sigma-Aldrich) in 0.1 % Tween-20 (Merck) PBS solution at room temperature in a humid chamber. Cells were then stained with primary antibodies against von Willebrand factor (vWf; Dako #A0082, RRID: AB_2,315,602), αSMA (Sigma-Aldrich #A5228, RRID: AB_262,054) or Vimentin (Progen #GP53, RRID: AB_2,687,459) over night at 4 °C in a humid chamber and then with secondary Alexa488- and Alexa546-conjugated antibodies for 1 hour at room temperature in a humid chamber in the dark. Nuclei were stained with 4′,6-diamidino-2-phenylindole (DAPI) for 10 min. Cells were covered with mounting medium (Leica) and microscopic images were obtained using a DM2000 LED microscope and Leica Application Suite v3.7.4 software (Leica).

### 2.5 Cell culture experiments

Adenosine 5′-(α,β-methylene)diphosphate (AMP-CP, a CD73 antagonist), 2′(3′)-*O*-(4-benzoylbenzoyl)adenosine 5′-triphosphate triethylammonium salt (BzATP, a metabolically stable ATP derivative), CGS-21,680 hydrochloride hydrate (CGS, an adenosine A_2A_ receptor agonist), BAY 60–6583 (BAY, an adenosine A_2B_ receptor agonist) and tumor necrosis factor alpha (TNFα) were purchased from Sigma-Aldrich. Sodium polyoxotungstate (POM-1, a nucleoside triphosphate diphosphohydrolase [NTPDase] inhibitor) was obtained from Tocris Bioscience and transforming growth factor beta1 (TGFβ1) was purchased from R&D Systems. All reagents were dissolved in PBS. Cells were seeded on a 1 % gelatin coated 6-well plate with 100.000 cells per well in 2 ml medium and grown to confluency at 37 °C and 5 % CO_2_. Confluent cells were treated with the following conditions: TGFβ1 (10 ng/ml), TNFα (5 ng/ml), POM-I (10 µM), BzATP (25 µM), AMP-CP (10 µM), CGS (10 µM), BAY (10 µM) for 14 days with medium changes every 2–3 days.

### 2.6 Western blot analysis

Proteins were extracted from cells using RIPA lysis buffer (Merck) supplemented with protease and phosphatase inhibitors (Roche). For protein separation, 7.5 µg of total protein was loaded on a 10 % polyacrylamide gel and subsequently blotted onto a nitrocellulose membrane using a tank blot setup (Bio-Rad). The total protein expression was analyzed after incubation with primary antibodies for CD31 (Cusabio #Pa851852), VE-cadherin (Abcam #33,168, RRID: AB_870,662), αSMA (Sigma-Aldrich #A5228, RRID: AB_262,054), GAPDH (Cell Signaling #2118, RRID: AB_561,053), β-Actin (Cell Signaling #4967, RRID: AB_330,288), p38 (Cell Signaling #8690 RRID: AB_10,999,090) and p-p38 (Cell Signaling #4511 RRID: AB_2,139,682) at 4 °C overnight. Secondary antibodies (Jackson Immuno #111–035–003 and #115–035–044) were subsequently incubated at room temperature for 1 hour. Bands were visualized using a chemiluminescence system (GE-Healthcare) and intensities were quantified with ImageJ software (NIH).

### 2.7 Semi-quantitative real-time PCR (qPCR)

Total RNA was extracted from cells using RNeasy Mini Kit (Qiagen) and the obtained RNA was reverse transcribed using Quantitect Reverse Transcription Kit (Qiagen). Kits were used according to manufacturer’s instructions. Quantitative real-time PCR was performed using Promega GoTaq PCR Master Mix (Promega) on a StepOnePlus real-time cycler (Applied Biosystems) with primers against *SNAI1* (5′-CACTATGCCGCGCTCTTT-3′, 3′- TAGGGCTGCTGGAAGGTAAA-5′), *SNAI2* (5′- GCCAAACTACAGCGAACTGG-3′, 3′- CACAGTGATGGGGCTGTATG-5′), *TWIST1* (5′- GGCTCAGCTACGCCTTCTC-3′, 3′- TCCTTCTCTGGAAACAATGACA-5′), *ZEB1* (5′- AACCCAACTTGAACGTCACA-3‘, 3′- TTACACCCAGACTGCGTCAC-5‘), *ZEB2* (5′- AACAAGCCAATCCCAGGAG-3‘, 3′-GTTGGCAATACCGTCATCCT-5′), *COL3A1* (5′-TGGAGGATGGTTGCACGAAA-3′, 3′-ACAGCCTTGCGTGTTCGATA-5′), MMP2 (5′- ACATCAAGGGCATTCAGGAG-3′, 3′-GCAGATCTCAGGAGTGACAGG-5′), *FN1* (5′- CCAAGGCTGGATGGTAG-3′, 3′- TTGTGTCCTGATCGTTGCAT-5′) and *CDH2* (5′- TCACTGCTCAGGACCCAGAT-3′, 3′-CCAATTGGCAGGATCAGATAA-5′). Expression levels of *GAPDH* (5′- CTGCACCACCAACTGCTTAG-3′, 3′- ACAGTCTTCTGGGTGGCAGT-5′) were used as a reference to normalize the obtained results using the ∆∆C_T_ method.

### 2.8 ATP content assay

Cell culture and VEC treatment were performed as described in Section 2.4. For the ATP measurement, 100 µL of the culture medium from each well was transferred to an opaque 96-well plate and mixed with 100 µL of a luminescence-based ATP detection reagent (CellTiter-Glo® 2.0, Promega). After incubation for 10 minutes at room temperature, the ATP content was determined by measuring luminescence with a microplate reader (FLUOstar Omega, BMG LABTECH).

### 2.9 Statistical analysis

Data are presented as mean ± standard error of mean (SEM). Statistical analysis and creation of graphs were performed using GraphPad Prism 6 (GraphPad Software, La Jolla, CA, USA). Comparisons between groups were determined using *Kruskal-Wallis* test with *Dunn’s multiple comparisons*. A p-value <0.05 was considered significant.

## 3 Results

### 3.1 Primary human VEC undergo EndMT after treatment with TGFβ or TNFα

**Fig. 1:**
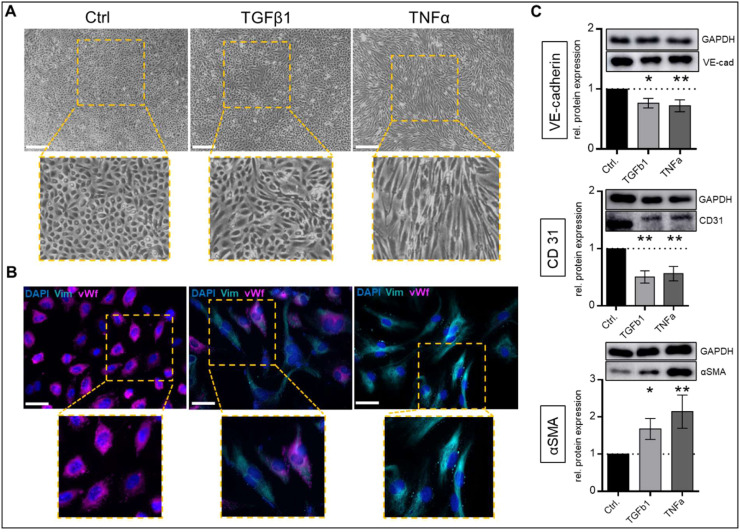
**EndMT induction in VEC**. Cells were incubated with 10 ng/ml transforming growth factor beta (TGFβ1), 5 ng/ml tumor necrosis factor alpha (TNFα) or without treatment (Ctrl) for 14 days. Representative light microscopic assessment of cell shape (scale bars 500 µm, A). Representative immunofluorescent staining of vimentin (Vim, cyan) and von Willebrand factor (vWF, magenta), nuclei were counterstained with 4′,6-diamidino-2-phenylindole (DAPI, blue; scale bars 50um, B). Analysis of protein expression by western blotting for vascular endothelial cadherin (VE-cadherin), cluster of differentiation 31 (CD31) and alpha smooth muscle actin (αSMA; C) normalized to Glyceraldehyde 3-phosphate dehydrogenase (GAPDH). Data are presented as means ± SEM; n = 11; Analysis was performed by Kruskal-Wallis test *p < 0.05 **p < 0.01 ***p < 0.001.

TGFβ and TNFα are well-established inducers of EndMT [31,32]. Treatment with TGFβ for 14 days resulted in elongated cell shape with decreased vWf expression and increased vimentin expression compared to controls (Fig. 1A/B) as demonstrated by immunofluorescent staining. Protein expressions of additional endothelial markers CD31 and VE-cadherin were significantly reduced (CD31: p < 0.01, VE-cadherin: p < 0.05; Fig. 1C) while the mesenchymal marker αSMA was increased (p < 0.05; Fig. 1C). TNFα treatment resulted in a more definite morphological change to spindle-shaped cell bodies with completely absent vWf signal and strong vimentin expression (Fig. 1A/B). Simultaneously, CD31 and VE-cadherin protein expression was significantly decreased (p < 0.01; Fig. 1C) and αSMA was increased (p < 0.01; Fig. 1C) upon TNFα treatment.

### 3.2 Enhanced ATP impact promotes mesenchymal phenotype in VEC

**Fig. 2:**
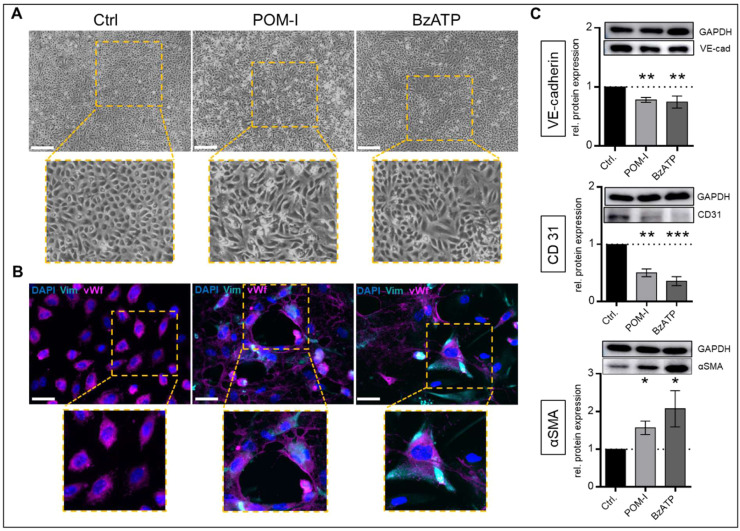
**Enhanced ATP impact promotes mesenchymal phenotype in VEC.** Cells were incubated with 10 µM Sodium polyoxotungstate (POM-1, a nucleoside triphosphate diphosphohydrolase [NTPDase] inhibitor), 25 µM 2′(3′)-*O*-(4-benzoylbenzoyl)adenosine 5′-triphosphate triethylammonium salt (BzATP, a prototypic P2X receptor agonist) or without treatment (Ctrl) for 14 days. Representative light microscopic assessment of cell shape (scale bars 500um, A). Representative immunofluorescent staining of vimentin (Vim, cyan) and von Willebrand factor (vWF, magenta), nuclei were counterstained with 4′,6-diamidino-2-phenylindole (DAPI, blue; scale bars 50um, B). Analysis of protein expression by western blotting for vascular endothelial cadherin (VE-cadherin), cluster of differentiation 31 (CD31) and alpha smooth muscle actin (aSMA; C) normalized to Glyceraldehyde 3-phosphate dehydrogenase (GAPDH). Data are presented as means ± SEM; n = 11; Analysis was performed by Kruskal-Wallis test *p < 0.05 **p < 0.01 ***p < 0.001.

In the purinergic signaling cascade, hydrolysis of ATP to AMP is catalyzed by the ectonucleotidase CD39. Inhibition of CD39 by POM-I treatment significantly altered the morphology of VEC to an elongated, almost ramified appearance compared to controls (Fig. 2A). Expression of vWf was less prominent than in untreated cells and vimentin expression was detectable by immunofluorescent staining (Fig. 2B). Endothelial markers CD31 and VE-cadherin were significantly decreased (p < 0.01; Fig. 2C) while αSMA protein expression was significantly increased compared to controls (p < 0.05; Fig. 2C) as shown by western blotting. To assess whether the distinct effects of POM-I treatment are related to elevated ATP, BzATP, a prototypic P2X receptor agonist with 5- to 10-fold higher potency than ATP [33], was used. VEC treated with BzATP showed similar morphological alterations and pattern of vWf and vimentin expression as compared to POM-I treatment (Fig. 2A/B). Protein expressions of CD31, VE-cadherin (p < 0.01) and αSMA (p < 0.05) were also significantly altered equal to POM-I treatment with a more distinct decrease in CD31 (p < 0.001; Fig. 2C).

### 3.3 Inhibition of CD73 but also adenosine receptor activation promotes mesenchymal phenotype in VEC

**Fig. 3:**
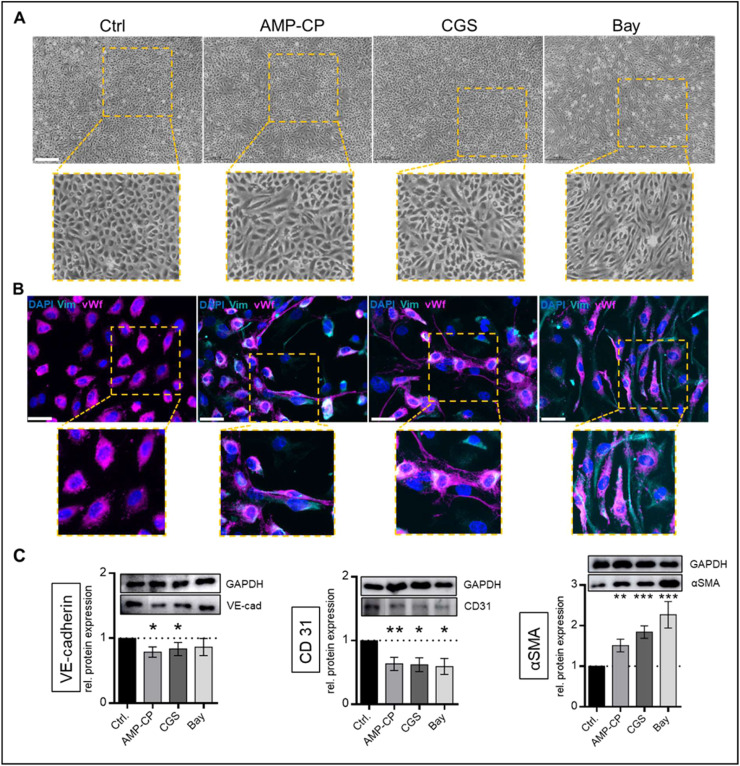
**Inhibition of CD73 but also adenosine receptor activation promotes mesenchymal phenotype in VEC**. Cells were incubated with 10 µM Adenosine 5′-(α,β-methylene)diphosphate (AMP-CP, a CD73 antagonist), 10 µM CGS-21,680 hydrochloride hydrate (CGS, an adenosine A_2A_ receptor agonist), 10 µM BAY 60–6583 (Bay, an adenosine A_2B_ receptor agonist) or without treatment (Ctrl) for 14 days. Representative light microscopic assessment of cell shape (scale bars 500um, A). Representative immunofluorescent staining of vimentin (Vim, cyan) and von Willebrand factor (vWF, magenta), nuclei were counterstained with 4′,6-diamidino-2-phenylindole (DAPI, blue; scale bars 50um, B). Analysis of protein expression by western blotting for vascular endothelial cadherin (VE-cadherin), cluster of differentiation 31 (CD31) and alpha smooth muscle actin (aSMA; C) normalized to Glyceraldehyde 3-phosphate dehydrogenase (GAPDH). Data are presented as means ± SEM; n = 11; Analysis was performed by Kruskal-Wallis test *p < 0.05 **p < 0.01 ***p < 0.001.

Dephosphorylation of AMP to adenosine is mediated by CD73. Inhibition of the endogenous adenosine formation by AMP-CP resulted in a spindle shape morphology in treated VEC with stable vWf expression while Vimentin was detectable via immunofluorescent staining as compared to controls (Fig. 3A/B). Protein expressions of additional endothelial markers VE-cadherin and CD31 were significantly reduced (VE-cadherin: p < 0.05, CD31: p < 0.01; Fig. 3C) and αSMA expression was increased (p < 0.01; Fig. 3C). Interestingly, treatment with CGS or BAY, specific agonists of the A_2A_ and A_2B_ adenosine receptors, resulted in similar effects. Cell morphology was altered in a ramified (CGS) and elongated (BAY) manner with slightly more distinct Vimentin expression in BAY-treated VEC (Fig. 3A/B). CD31 protein expression was also significantly reduced (p < 0.05) while αSMA expression was increased (p < 0.001) compared to controls (Fig. 3C). VE-cadherin expression was decreased as well by CGS treatment (p < 0.05) but failed to reach statistical significance with BAY treatment (Fig. 3C).

### 3.4 TGFβ stimulation, inhibition of CD39 or treatment with BzATP enhances p38 phosphorylation in VEC

**Fig. 4:**
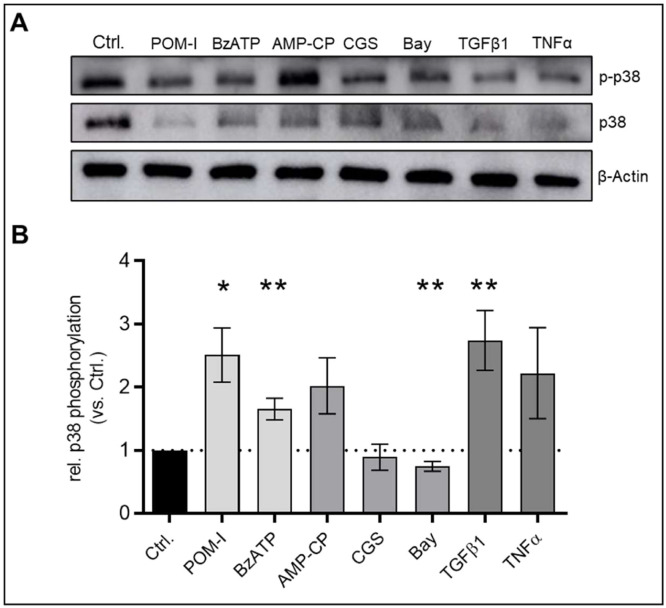
**TGFβ stimulation, inhibition of CD39 or treatment with BzATP enhances p38 phosphorylation in VEC.** Cells were incubated with 10 ng/ml transforming growth factor beta (TGFβ), 5 ng/ml tumor necrosis factor alpha (TNFα), 10 µM Sodium polyoxotungstate (POM-1, a nucleoside triphosphate diphosphohydrolase [NTPDase] inhibitor), 25 µM 2′(3′)-*O*-(4-benzoylbenzoyl)adenosine 5′-triphosphate triethylammonium salt (BzATP, a prototypic P2X receptor agonist), 10 µM Adenosine 5′-(α,β-methylene)diphosphate (AMP-CP, a CD73 antagonist), 10 µM CGS-21,680 hydrochloride hydrate (CGS, an adenosine A_2A_ receptor agonist), 10 µM BAY 60–6583 (Bay, an adenosine A_2B_ receptor agonist) or without treatment (Ctrl) for 14 days. Analysis of protein expression by western blotting for p38 MAPK (p38) and its phosphorylated form (p-p38), representative blots are shown (A), values are displayed as ratio of p-p38 per p38 relative to control. β-Actin was utilized as a housekeeping protein for normalization. Data are presented as means ± SEM; n = 5; Analysis was performed by Kruskal-Wallis test *p < 0.05 **p < 0.01 ***p < 0.001.

Activation of p38 MAPK (p38) has been linked to TGFβ1-induced EMT in human renal epithelial cells [34] as well as to EndMT in human dermal microvascular endothelial cells [35]. Therefore, we investigated p38 MAPK phosphorylation (p-p38) in VEC after EndMT induction. Relative protein expression levels of p-p38 were significantly elevated in cells after TGFβ1 treatment (p < 0.01) as well as after CD39 inhibition by POM-I (p < 0.05) or treatment with BzATP (p < 0.01), however this effect was not observed when CD73 was inhibited by AMP-CP (Fig. 4). Stimulation of VEC with TNFα or activation of purinergic receptors with CGS did not significantly alter p38 phosphorylation while treatment with BAY significantly decreased phosphorylation (p < 0.01) as demonstrated by Western blot analysis (Fig. 4).

### 3.5 Inhibition of key enzymes alters EndMT associated transcription factor expression in VEC

**Fig. 5:**
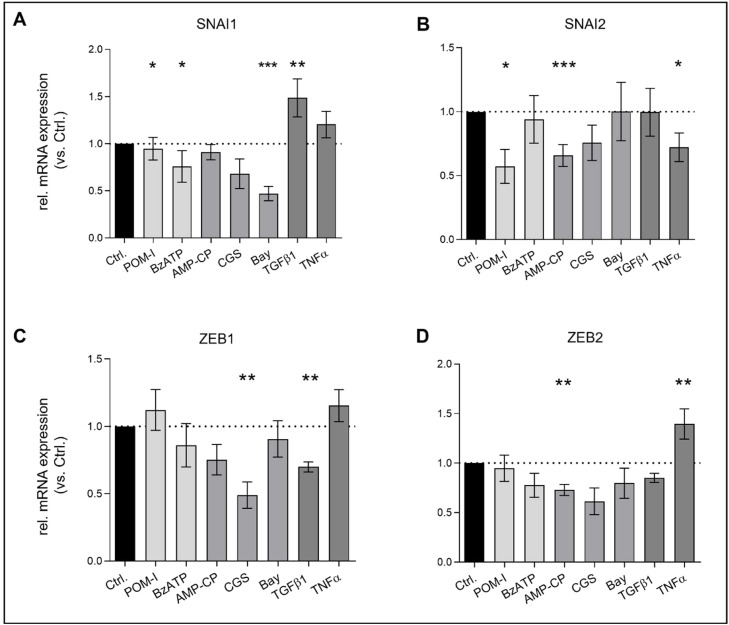
**Inhibition of key enzymes alters EndMT-associated transcription factor expression in VEC.** Cells were incubated with 10 ng/ml transforming growth factor beta (TGFβ), 5ng/ml tumor necrosis factor alpha (TNFα), 10 µM Sodium polyoxotungstate (POM-1, a nucleoside triphosphate diphosphohydrolase [NTPDase] inhibitor), 25 µM 2′(3′)-*O*-(4-benzoylbenzoyl)adenosine 5′-triphosphate triethylammonium salt (BzATP, a prototypic P2X receptor agonist), 10 µM Adenosine 5′-(α,β-methylene)diphosphate (AMP-CP, a CD73 antagonist), 10 µM CGS-21,680 hydrochloride hydrate (CGS, an adenosine A_2A_ receptor agonist), 10 µM BAY 60–6583 (Bay, an adenosine A_2B_ receptor agonist) or without treatment (Ctrl) for 14 days. Analyses of mRNA expression by semi-quantitative real-time PCR are shown. GAPDH was utilized as a housekeeping gene for normalization. Data are presented as means ± SEM; n = 5; Analysis was performed by Kruskal-Wallis test *p < 0.05 **p < 0.01 ***p < 0.001.

Many transcription factors have been associated with EndMT, especially after TGFβ induction. Amongst them, the snail family of transcription factors (SNAIL, *SNAI1*; SLUG, *SNAI2*) is the most intensely studied [36,37]. Others, such as *ZEB1/ZEB2,* play equally important roles in EndMT and cardiovascular diseases but are rather associated with TNFα induction [38,39]. Hence, we explored the gene expression of these regulating factors in VEC after EndMT induction. As expected, TGFβ1 stimulation resulted in a significantly increased expression of *SNAI1* (p < 0.01; Fig.5A) while VEC after TNFα induction showed elevated expression of *ZEB1 (ns)* and *ZEB2* (p < 0.001), respectively (Fig. 5C/D). Interestingly, inhibition of CD39 resulted in significantly decreased expression of both SNAIL family transcription factors as compared to controls (POM-I p < 0.05; Fig. 5A/B), whereas treatment with BzATP exclusively led to *SNAI1* downregulation (p < 0.05; Fig. 5A). Furthermore, inhibition of CD73 resulted in significant downregulation of *SNAI2* (p < 0.001; Fig. 5B) and *ZEB2* (p < 0.01; Fig. 5D). In addition, A_2A_ receptor activation via CGS treatment led to significantly decreased *ZEB1* (p < 0.01; Fig. C) expression and A_2B_ receptor activation via BAY to significant downregulation of *SNAI1* (p < 0.001; Fig. 5A).

### 3.6 Patient characteristics

Patient characteristics of the included donors are summarized in Table 1. In total, 11 donors were analyzed. The majority of donors were male (73 %), whereas 27 % were female. Mean age was 60 ± 13 years and mean body mass index was 28 ± 5 kg/m². No significant differences were observed with respect to age or body mass index. Heart transplantation represented the most common primary indication (73 %), followed by valve replacement procedures (27 %). Among cardiovascular risk factors, arterial hypertension was most frequently present (45 %), while smoking and dyslipoproteinemia were each documented in 9 % of donors. Regarding comorbidities, chronic kidney disease was present in 27 % of donors and coronary artery disease in 18 %, whereas no extracardiac vascular diseases were reported. Diabetes mellitus was documented in one donor (9 %). Medication use included predominantly diuretics (73 %), anticoagulants (55 %), and statins (36 %). ACE inhibitors and β-adrenergic receptor antagonists were each administered in 27 % of donors. Several medication-related parameters showed statistically significant differences, as detailed in Table 1. Laboratory analysis revealed a mean platelet count of 222 ± 46 × 10³/µl and a mean leukocyte count of 8 ± 4 × 10³/µl. Mean C-reactive protein levels were 2 ± 2 mg/dl. Platelet count, leukocyte count, and C-reactive protein levels showed statistically significant differences (Table 1).

**Table 1:** Patient characteristics.

	Patient number	*P* value
Donor [number], n	11	
Sex [female], n (%)	3 (27)	0,0816
Sex [male], n (%)	8 (73)	0,0004
Age [years]	60 ± 13	n.d.
Body Mass Index [kg/m^2^]	28 ± 5	0,1751
Primary Indication, n (%)		
Heart Transplantation	8 (73)	0,0004
Valve Replacement	3 (27)	0,0816
Cardiovascular Risk Factors, n(%)		
Smoking	1 (9)	0,3409
Arterial Hypertension	5 (45)	0,0162
Dyslipoproteinemia	1 (9)	0,3409
Comorbidities, n (%)		
Chronic Kidney Disease	3 (27)	0,0816
Coronary Artery Disease	2 (18)	0,1669
Extracardiac Vascular Diseases	0	n.a.
Diabetes Mellitus	1 (9)	0,3409
Medication, n (%)		
Statins	4 (36)	0,0379
Diuretics	8 (73)	0,0004
ACE-Inhibitors	3 (27)	0,0816
β-adrenergic receptor antagonists	3 (27)	0,0816
Anticoagulants	6 (55)	0,0061
Laboratory Values		
Platelets [x1000/µl]	222 ± 46	<0,0001
Leukocytes [x1000/µl]	8 ± 4	0,0158
C-Reactive Protein [mg/dl]	2 ± 2	<0,0001
ACE, angiotensin-converting enzyme; n.d., normally distributed; BMI, body mass index; n.a., not applicable; Categorical variables are presented as the number of affected donors (n) and the proportion of the total cohort (%), while continuous variables are reported as mean ± standard error of the mean (SEM). Continuous variables were first tested for normal distribution using the D’Agostino–Pearson omnibus normality test and subsequently compared, as were the remaining parameters, using a one-sample *t*-test. Body mass index and laboratory values were analyzed in comparison to established upper reference values.		

## 4 Discussion

Despite extensive research on EndMT in cardiovascular diseases [[40], [41], [42]] and on purinergic signaling in cardiovascular pathologies [19,20,43], their potential interconnection remains largely unexplored. In a previous study from our group, we demonstrated enhanced biomineralization in VIC by inhibition of CD39 and ENPP1, whereas inhibition of CD73 resulted in protective effects [21]. Recent studies have also shown that VEC are able to actively induce pathological remodeling and calcification in VIC via the process of EndMT [15] and, in reverse, that VIC can inhibit EndMT in VEC [16]. Considering the profound effects of the purinergic signaling system on VIC and the extensive crosstalk between VEC and VIC via EndMT in pathobiological processes, unraveling a potential link may lead to new therapeutic approaches in the field. In this study, we isolated primary human VEC and subjected the cells to inhibitors and agonists of the purinergic signaling system to evaluate the effect on EndMT induction. We found that VEC from all donors maintained a predominantly endothelial phenotype prior to experimental stimulation (Supplementary Fig. 1) and were generally capable of undergoing EndMT under similar conditions. Treatment with TGFβ1 or TNFα served as a baseline reference, as these are well-established inducers of EndMT [31,32]. Inhibition of the key enzymes CD39, ENPP1 and CD73, as well as activation of purinergic A_2_ and P_2_ receptors leads to a mesenchymal phenotype in these VEC but first explorations of the underlying mechanisms suggest different processes.

Inhibition of the ATP-converting enzymes CD39 and ENPP1 increases ATP availability (Supplementary Fig. 2), which leads to higher activation of purinergic receptors. The rates of extracellular ATP and AMP hydrolysis have been shown to be comparable between VIC and VEC [44]. Inhibition of the ATP-converting enzymes with POM-I leads to a mesenchymal phenotype and EndMT-associated protein expression in VEC in the present study. This finding was further supported by experimental treatment with BzATP, an ATP analog and partial ATP receptor agonist. Although similar effects on VEC morphology and protein expression indicate an important role of elevated extracellular ATP availability, the underlying mechanisms seem to differ. Activation of p38 MAPK plays a crucial role in endothelial cell activation, pathogenesis of atherosclerosis and has been linked to EndMT in human dermal microvascular endothelial cells [35,45]. In our experiments, treatment with POM-I but not the administration of BzATP leads to increased p38 phosphorylation in VEC. Interestingly, regulation of EndMT-associated transcription factors of the snail family was different between the treatments as well. BzATP administration led to decreased *SNAI1* gene expression, whereas POM-I treatment caused decreased *SNAI2* gene expression in VEC. Compared to EndMT induction with the well-established inducers TGFβ1 or TNFα, CD39 and ENPP1 inhibition and TGFβ1 treatment have similar effects on p38 phosphorylation but decreased *SNAI2* expression is similar to TNFα induction. However, BzATP treatment caused reduced expression of *SNAI1*. In our experiments, the generally observed decrease in gene expression of snail family transcription factors might be due to an already proceeded negative feedback loop and indicates that EndMT induction by inhibition of CD39 and ENPP1, as well as BzATP treatment occurs in a different time-dependent course. Loss-of-function mutations of ENPP1 are known to lead to generalized arterial calcification of infancy and P2Y receptor activation is also linked to vascular calcification [[46], [47], [48]]. Our data now suggest that EndMT may play a crucial role in these processes and contribute to CAVD.

In addition to our findings on the inhibition of CD39 and enhanced extracellular ATP availability, we examined the effects of altered extracellular adenosine availability. The enzyme CD73 hydrolyzes the conversion of AMP to adenosine, thus generally leading to higher extracellular AMP and lower adenosine availability with activation of adenosine receptors [49]. In our experiments, inhibition of CD73 with the inhibitor AMP-CP also caused EndMT-associated morphological changes and alterations in protein expression of VEC. Interestingly, treatment with A_2A_ receptor agonist CGS or A_2B_ receptor agonist BAY led to a similar EndMT pattern with the most prominent morphological changes after BAY administration. The phosphorylation of p38 MAPK was not altered by adenosine receptor activation but increased by trend after inhibition of CD73. Furthermore, *SNAI2* gene expression was decreased in VEC after AMP-CP treatment but not after adenosine receptor activation. These findings match our aforementioned observations after CD39 and ENPP1 inhibition and further indicate that inhibition of the key enzymes of the purinergic signaling system affects EndMT via other mechanisms compared to purinergic receptor activation. Similar to BzATP treatment, the administration of A_2B_ receptor agonist BAY regulated *SNAI1* gene expression, suggesting related mechanisms of action. However, A_2A_ receptor agonist CGS caused decreased *ZEB1* and *ZEB2* gene expression, which was also identified in TGFβ1-treated VEC. Collectively, the induction of EndMT via CD73 inhibition but also adenosine receptor activation may seem conflicting but recently it was shown that patients with CD73 deficiency exhibit an increased activity of tissue-nonspecific alkaline phosphatase, leading to a higher dephosphorylation of AMP to adenosine [50]. Considering this mechanism, we suggest that the observed effects are likely related to alternative adenosine production. While adenosine is considered anti-inflammatory and rather protective in immune-modulation and the myocardium [49,[51], [52], [53]], our data suggest that elevated extracellular adenosine availability has deleterious effects on VEC physiology. In a previously published study of our group, we demonstrated the diverse effects of the purinergic signaling system on the degeneration of VIC with a largely similar outcome [21], indicating that a dysregulation of the purinergic signaling system has profound effects on aortic valve biology and occurrence of calcific processes. Additional transcriptional analyses further suggested that different stimuli induce distinct EndMT-associated gene expression profiles in VECs. While TGFβ1 promoted a broader pro-fibrotic signature, TNFα primarily increased genes related to matrix remodeling, whereas purinergic modulation resulted in more intermediate responses (Supplementary Fig. 3).

Collectively, our study demonstrates for the first time that dysregulation of the purinergic signaling system promotes EndMT in human VECs, providing novel insights into aortic valve biology and identifying potential targets for pharmacological intervention. Our in vitro experiments reveal that altered purinergic signaling activates distinct intracellular pathways, depending on whether key enzymes (CD39, ENPP1, CD73) are inhibited or purinergic receptors are stimulated. These observations complement previous findings on VIC degeneration and suggest a critical interplay between the two major cell types of the aortic valve in response to dysregulated purinergic signaling. Future studies employing co-culture and 3D culture systems will more accurately mimic native valve conditions, offering clinically relevant insights. Further investigation is warranted to elucidate the crosstalk between VECs and VICs and to define the precise molecular mechanisms underlying purinergic regulation of EndMT.

## Funding sources

The work was funded by the Research Committee of the Medical Faculty of the Heinrich Heine University (Weber A. 2018).

## Data availability statement

The data that support the findings of this study are largely available in the Materials and Methods, Results, and/or Supplemental Material of this article or can be obtained from the corresponding author upon reasonable request.

## CRediT authorship contribution statement

**Vera Schmidt:** Investigation, Methodology, Formal analysis, Visualization, Writing – original draft. **Andreas Weber:** Conceptualization, Supervision, Funding acquisition, Project administration, Data curation, Validation. **Artur Lichtenberg:** Conceptualization, Resources, Writing – review & editing. **Jürgen Schrader:** Validation, Writing – review & editing. **Pascal Martsch:** Data curation, Validation, Formal analysis, Writing – review & editing. **Mareike Barth:** Data curation, Validation, Writing – review & editing. **Payam Akhyari:** Conceptualization, Data curation, Resources, Validation, Writing – review & editing.

## Declaration of competing interest

The authors declare the following financial interests/personal relationships which may be considered as potential competing interests:

Andreas Weber reports financial support was provided by Medical Faculty and University Hospital Düsseldorf, Heinrich-Heine-University. If there are other authors, they declare that they have no known competing financial interests or personal relationships that could have appeared to influence the work reported in this paper.

## Appendix Supplementary materials

Image, application 1
