# Supplementary material for: Purinergic signaling modulates endothelial-to-mesenchymal transition in human aortic valve endothelial cells
Source: BBA Adv. 2026 Apr 18;9:100190. doi: 10.1016/j.bbadva.2026.100190 (PMC13126352; doi:10.1016/j.bbadva.2026.100190)
Supplement: Supplementary file 1 [file mmc1.pdf]

Supplementary Material

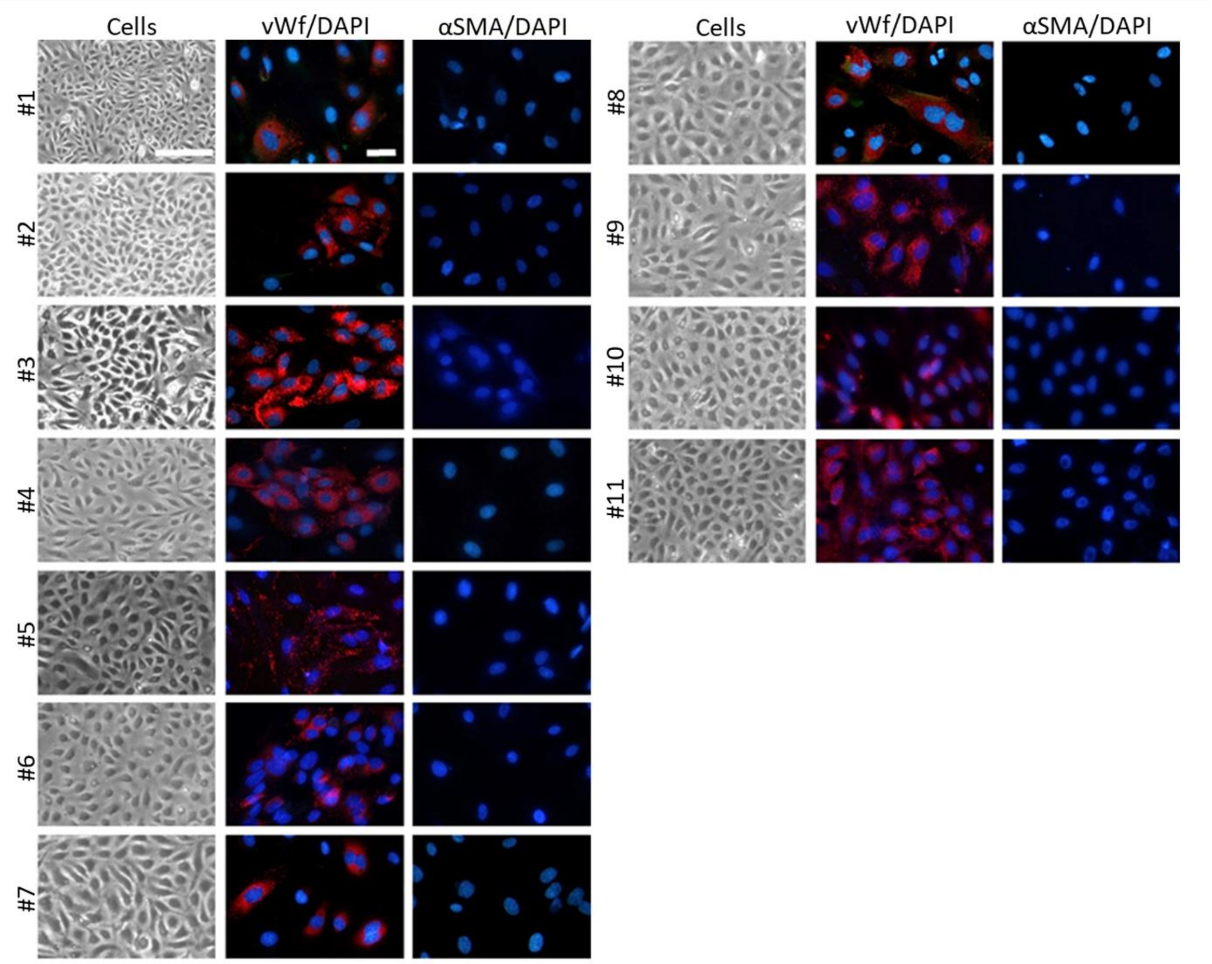

Supplementary Figure 1: Characterization of aortic valve endothelial cells.

Bright field microphotographs of VEC (Cells). Merged immunofluorescence images for endothelial marker VWF or mesenchymal marker αSMA and DAPI nuclear staining. Characterization was conducted for every single donor (#1-#11). Scale bars 100 µm (cells) and 50µm (vWf/aSMA/DAPI).

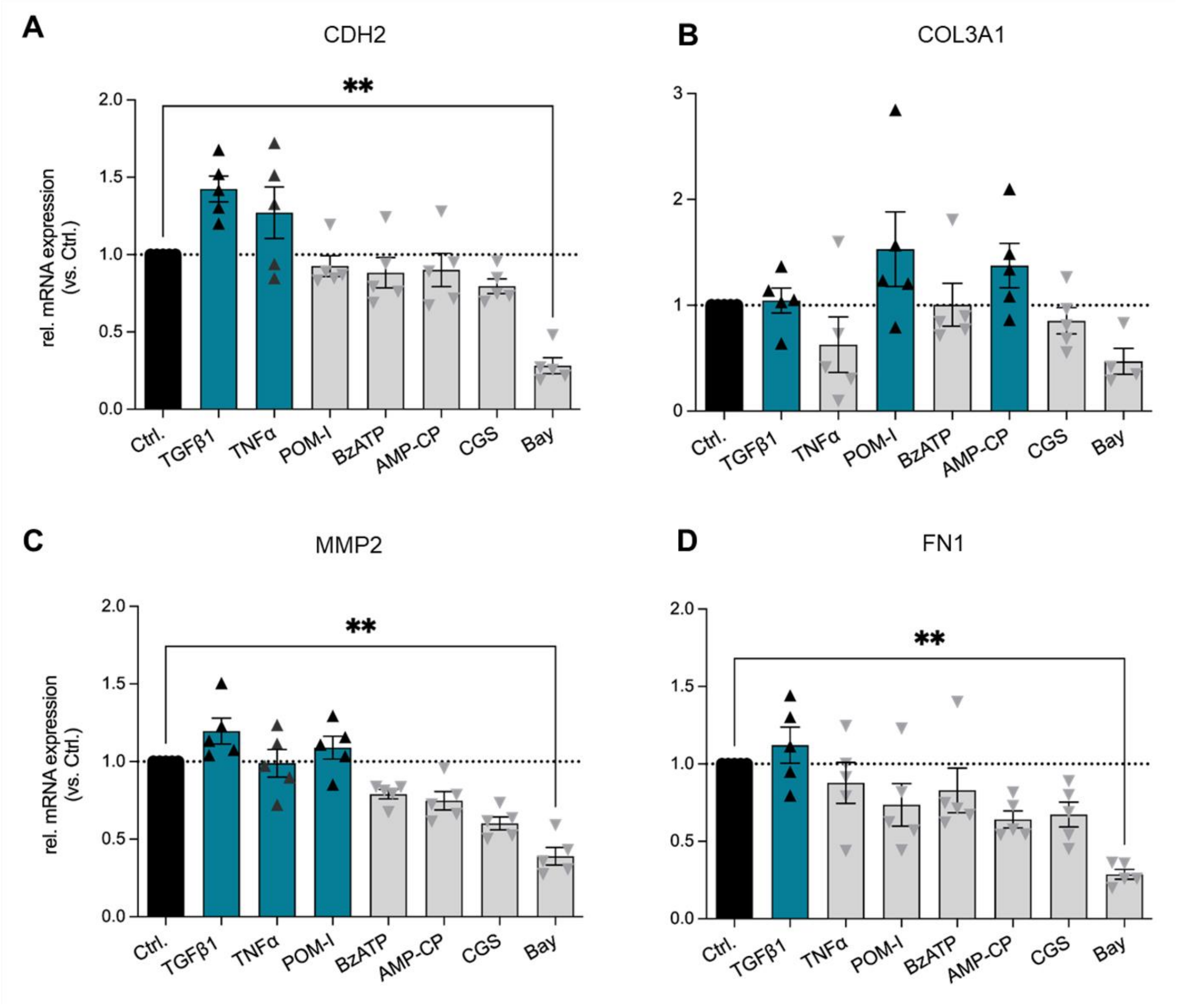

Supplementary Figure 2: Significant increase in ATP content after POM-I treatment of VEC.

Cells were incubated with 10 ng/ml transforming growth factor beta (TGFβ), 5 ng/ml tumor necrosis factor alpha (TNFα), 10 µM Sodium polyoxotungstate (POM-1, a nucleoside triphosphate diphosphohydrolase [NTPDase] inhibitor), 25 µM 2′(3′)-O-(4-benzoylbenzoyl)adenosine 5′-triphosphate triethylammonium salt (BzATP, a prototypic P2X receptor agonist), 10 µM Adenosine 5′-(α,β-methylene)diphosphate (AMP-CP, a CD73 antagonist), 10 µM CGS-21680 hydrochloride hydrate (CGS, an adenosine A2A receptor agonist), 10 µM BAY 60–6583 (Bay, an adenosine A2B receptor agonist) or without treatment (Ctrl) for 14 days. Analysis of ATP content relative to control conditions. Data are presented as means ± SEM; n=5; Analysis was performed by Kruskal-Wallis test; **p<0.01.

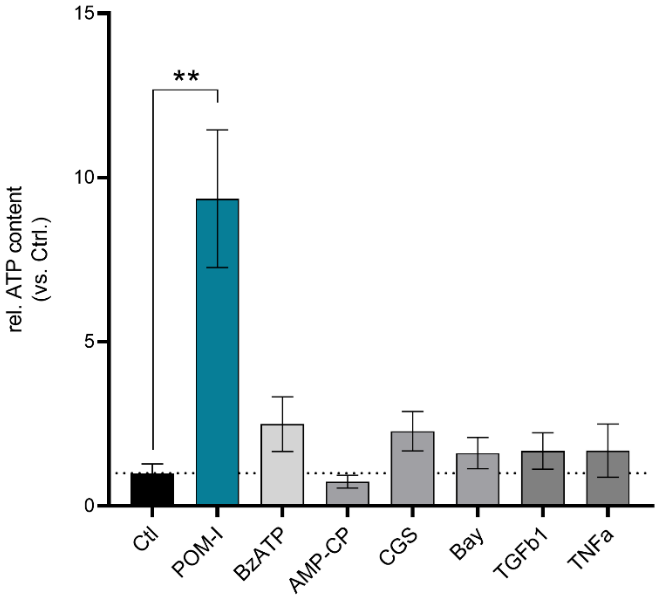

Supplementary Figure 3: Distinct EndMT-associated gene expression patterns in VECs after stimulation with TGFβ1, TNFα and purinergic modulators.

Cells were incubated with 10 ng/ml transforming growth factor beta (TGFβ), 5 ng/ml tumor necrosis factor alpha (TNFα), 10 µM Sodium polyoxotungstate (POM-1, a nucleoside triphosphate diphosphohydrolase [NTPDase] inhibitor), 25 µM 2′(3′)-O-(4-benzoylbenzoyl)adenosine 5′-triphosphate triethylammonium salt (BzATP, a prototypic P2X receptor agonist), 10 µM Adenosine 5′-(α,β-methylene)diphosphate (AMP-CP, a CD73 antagonist), 10 µM CGS-21680 hydrochloride hydrate (CGS, an adenosine A2A receptor agonist), 10 µM BAY 60–6583 (Bay, an adenosine A2B receptor agonist) or without treatment (Ctrl) for 14 days. Analyses of mRNA expression by semi quantitative real time PCR are shown. GAPDH was utilized as a housekeeping gene for normalization. Data are presented as means ± SEM; n=5; Analysis was performed by Kruskal-Wallis test; **p<0.01.
